# Preparation and Performance of 3D-Printed TiO_2_-Supported TPMS Structures for Photocatalytic Applications

**DOI:** 10.3390/molecules30193891

**Published:** 2025-09-26

**Authors:** Xi Chen, Chenxi Zhang, Xiao Chen, Ningning Li

**Affiliations:** School of Materials Science and Engineering, North China University of Water Resources and Electric Power, Zhengzhou 450045, China

**Keywords:** triply periodic minimal surface (TPMS) model, photocatalytic reactor, TiO_2_/PLA composite

## Abstract

This study addresses critical bottlenecks in photocatalytic water treatment technologies, including difficulties in recovering traditional powdered catalysts, low mass transfer efficiency in immobilized reactors, and limited structural diversity. By integrating topology optimization with 3D printing technology, we designed and fabricated five types of triply periodic minimal surface photocatalytic reactors (TPMS-PCRs) with hierarchical porous structures—Fischer-Radin-Dunn (FRD), Neovius (N), Diamond (D), I-graph Wrapped Package (IWP) and Gyroid (G). Using fused deposition modeling (FDM), these TPMS configurations were manufactured from polylactic acid (PLA), 1.5 wt% TiO_2_/PLA, and 2.5 wt% TiO_2_/PLA. The catalytic degradation performance of these structurally distinct reactors for organic pollutants varied significantly. Notably, the FRD-type TPMS-PCR loaded with 2.5 wt% TiO_2_ achieved a methylene blue (MB) degradation rate of 93.4% within 2.5 h under rotational flow conditions, compared to 87.5% under horizontal flow conditions.

## 1. Introduction

Semiconductor photocatalysis, particularly the use of titanium dioxide (TiO_2_) for water treatment, is considered a highly promising solution to environmental pollution problems. However, its transition from laboratory-scale research to large-scale practical application is hindered by several critical bottlenecks. Traditional nano-sized TiO_2_ powder catalysts, despite their high activity, suffer from issues such as agglomeration tendency, difficulty in recovery, potential secondary pollution, and low mass transfer efficiency in practical reactor systems [1,2,3]. Although immobilization techniques (e.g., loading catalysts onto flat plates or granular supports) partially address the recovery issue, they often come at the expense of the catalyst’s specific surface area and introduce new mass transfer limitations, resulting in reduced overall catalytic efficiency. Furthermore, conventional manufacturing processes restrict the complexity and design flexibility of reactor structures, making it challenging to optimize internal flow fields and light distribution within the reactor, which further limits performance improvement [4,5].

The emergence of additive manufacturing (3D printing) technology offers a revolutionary approach to overcoming the above challenges. Among various 3D printing processes, Fused Deposition Modeling (FDM) has shown great potential in fabricating functional devices due to its operational simplicity, low cost, and broad material compatibility [6,7,8]. For particulate materials lacking viscosity, such as catalysts, FDM technology can employ thermoplastic polymers (e.g., PLA, ABS) as support materials and binders. By pre-mixing catalyst particles with polymer pellets and feeding them into a screw extruder, the polymer melts at high temperatures and encapsulates the catalyst particles, enabling co-extrusion to ultimately produce monolithic catalysts with complex three-dimensional structures [9]. This method not only achieves effective catalyst immobilization but also provides a high degree of design freedom for the carrier structure.

In recent years, researchers have increasingly explored the application of FDM in the field of catalysis. For example, Binetti et al. [10] used PET polymer to fabricate monolithic supports with customized internal channels via FDM technology and systematically studied the impact of their structure on NOx removal performance. More recently, Liu et al. [11] developed an innovative strategy: first embedding chlorella powder into an ABS/TPU matrix and forming a porous scaffold via FDM, followed by subsequent treatment to successfully grow Fe_2_O_3_ on the scaffold surface, effectively addressing the immobilization and active site exposure issues of powder catalysts.

However, many existing 3D-printed photocatalytic reactors still rely on relatively conventional geometries (e.g., fixed-bed, annular, tubular, or rotating disk) [12,13,14,15], which struggle to synergistically optimize the three critical performance indicators: light distribution, mass transfer, and active site density.

**Figure 1:**
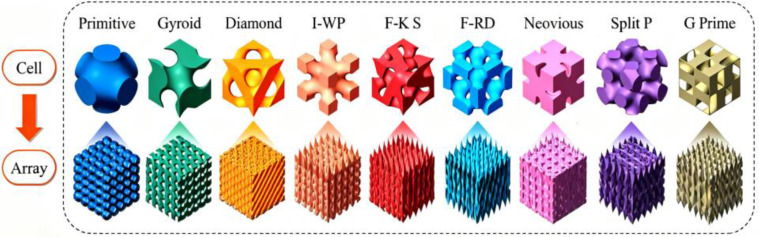
TPMS Types and Corresponding Unit Cells reprinted with permission from Ref. [20]. Copyright 2025 Elsevier Ltd.

To overcome these limitations and fully leverage the design freedom offered by 3D printing, this study introduces a new paradigm by integrating Triply Periodic Minimal Surfaces (TPMS) topology with one-step FDM fabrication of catalyst-embedded monolithic structures. TPMS are abstract, mathematically defined structures (e.g., Gyroid (G), Diamond (D), and Fischer-Radin-Dunn (FRD)) that exhibit periodicity in all three spatial dimensions and maintain zero mean curvature [16,17,18,19]. Figure 1 shows the TPMS types and their corresponding unit cells. They translate mathematical elegance into exceptional engineering performance: offering extremely high specific surface area, fully interconnected channels (ensuring excellent mass transfer), self-supporting robust structures, and highly tunable geometric parameters [20]. These inherent properties make TPMS structures ideal candidates for next-generation photocatalytic reactors. However, compared to their applications in other fields such as biomedicine or heat exchange, this potential remains underexplored.

This study used TiO_2_/PLA composite material, synthesized from nano-titanium dioxide and the thermoplastic polymer polylactic acid (PLA), as the printing feedstock for Fused Deposition Modeling (FDM). Five types of triply periodic minimal surface (TPMS) function models, including FRD, N, D, G, and IWP, were selected for investigation. The geometric node flowchart of the TPMS structures was designed using the 3D modeling software Blender (version 4.4), enabling the fabrication of TPMS-based photocatalytic reactors (TPMS-PCRs) via FDM 3D printing. The synthesized TPMS-PCRs were characterized by XRD, FTIR, and SEM/EDS. Furthermore, the degradation performance of the FDM-printed TPMS-PCRs was systematically evaluated by examining different flow field conditions and varying TiO_2_ catalyst loading levels.

This work demonstrates significant advancements over previous studies: (1) Employing a one-step FDM process to directly fabricate ready-to-use, TiO_2_-embedded monolithic TPMS reactors (TPMS-PCRs), eliminating the need for post-printing catalyst deposition and enhancing operational stability; (2) Systematically designing and comparing five distinct TPMS architectures (FRD, N, D, IWP, G) to elucidate the profound impact of topology on photocatalytic performance, moving beyond the study of single or conventional geometries; (3) Establishing clear structure-performance relationships by evaluating these reactors under different hydrodynamic conditions (horizontal vs. rotational flow fields), providing valuable insights for future reactor design and optimization.

We anticipate that this integrated strategy of topology optimization and additive manufacturing will open new avenues for constructing high-performance, customizable photocatalytic reactors, advancing the development of sustainable environmental remediation technologies.

## 2. Results and Discussion

### 2.1. Structural and Morphological Analysis

#### 2.1.1. X-Ray Diffraction

X-ray diffraction (XRD) was characterized on a Bruker D8 Advance diffractometer (Bruker, Berlin, Germany) using Cu Kα (1.5418 nm).

**Figure 2:**
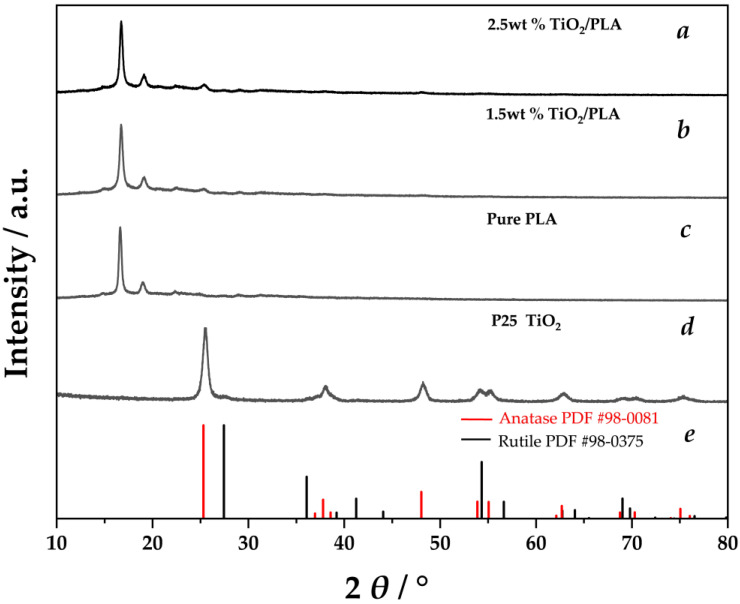
XRD pattern of the reactor printed using 2.5 wt% TiO_2_/PLA (**a**), 1.5 wt% TiO_2_/PLA (**b**), pure PLA (**c**) as raw material, TiO_2_ powder (**d**) and PDF card of TiO_2_ (anatase and rutile phase) (**e**).

As shown in Figure 2, the XRD pattern of TiO_2_ (P25, Shanghai Macklin Biochemical Technology Co., Ltd., Shanghai, China) confirms that it exists as a mixture of anatase (PDF #98-0081) and rutile (PDF #98-0375). The XRD patterns of reactors fabricated with 1.5 wt% TiO_2_/PLA and 2.5 wt% TiO_2_/PLA composites reveal them to be mixtures of PLA and TiO_2_. Furthermore, with increasing TiO_2_ doping content, the characteristic peak at about 25° assigned to TiO_2_ exhibits a corresponding increase in intensity. The slight shift of the diffraction peaks toward lower angles compared to pure TiO_2_ may be attributed to the presence of residual stress in the sample, resulting in an expansion of the interplanar spacing. The crystallite sizes of TiO_2_ in 1.5 wt% TiO_2_/PLA and 2.5 wt% TiO_2_/PLA, calculated using the Scherrer formula, were 15.12 nm and 16.97 nm, respectively, which are slightly smaller than that of the raw TiO_2_ (21.21 nm).

Under the assumption that the percentage of crystalline phases is proportional to the sum of the integral intensities of their diffraction peaks, the Easy Quantitative Analysis module in Jade 9 software was employed to analyze the deconvoluted peaks and fitted XRD patterns. The relative crystallinity of each phase in the samples was calculated based on the ratio of the integral intensity of each phase’s characteristic peaks to the total integral intensity of all peaks. Results indicate that the relative crystallinity of TiO_2_ in reactors prepared with 1.5 wt% TiO_2_/PLA and 2.5 wt% TiO_2_/PLA composites is 1.4 wt% and 2.3 wt%, respectively, which is in good agreement with the initial feeding ratios. The relative crystallinity values for each phase in the samples are summarized in Table A2.

#### 2.1.2. Contact Angle Test

The contact angles of PLA and TiO_2_/PLA composites were measured using an SDC-100 contact angle analyzer to evaluate their wettability. As shown in Table 1, the contact angle of pure PLA was 80.771°, indicating its hydrophobic nature. The incorporation of TiO_2_ nanoparticles, which are rich in surface hydroxyl groups (-OH), enhanced the hydrophilicity of the composites. With increasing TiO_2_ content, more hydrophilic -OH groups were introduced, leading to a reduction in surface energy. Furthermore, the presence of TiO_2_ nanoparticles increased the surface roughness, which also contributed to the improved hydrophilicity of the TiO_2_/PLA composites. The consistent contact angle values between the left and right sides suggest a uniform dispersion of TiO_2_ within the PLA matrix and demonstrate the high reproducibility of the sample preparation process.

#### 2.1.3. SEM/EDS

The morphology of the photocatalytic reactor was observed using a Hitachi SU 8010(Hitachi High-Tech Corporation, Tokyo, Japan) high-resolution field emission scanning electron microscope (SEM). Simultaneously, qualitative and semi-quantitative elemental analysis of the target sample was performed using energy dispersive spectroscopy (EDS).

**Figure 3:**
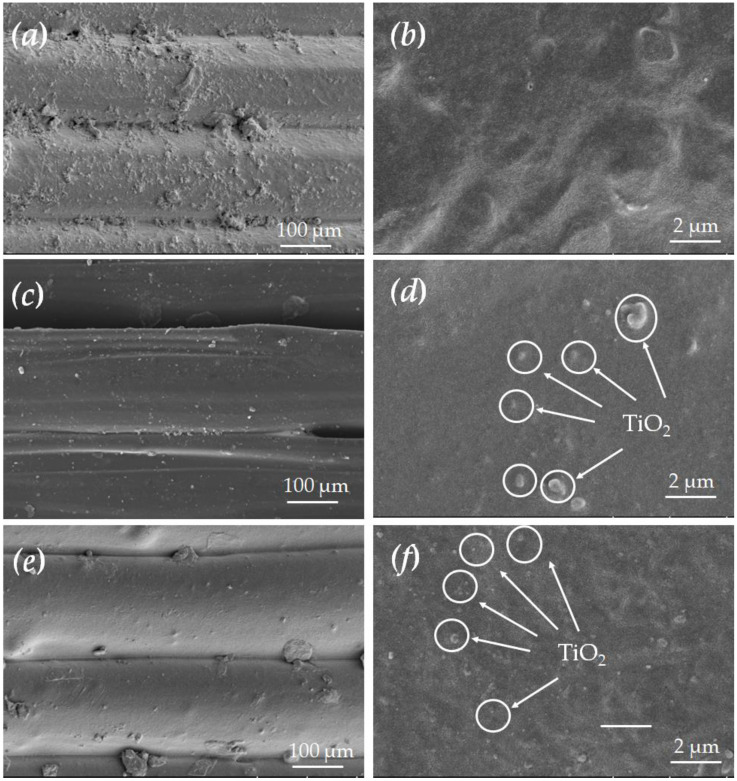
SEM of N-PCR surface ((**a**,**b**): fabricated using pure PLA; (**c**,**d**): fabricated using 1.5 wt% TiO_2_/PLA; (**e**,**f**): fabricated using 2.5 wt% TiO_2_/PLA).

**Figure 4:**
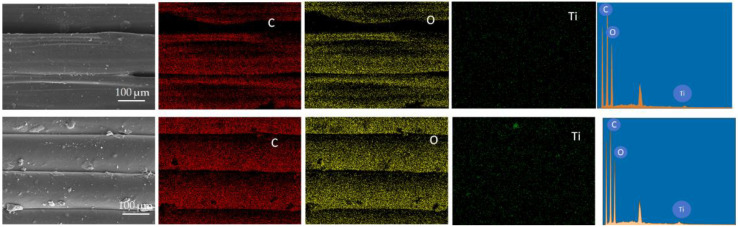
EDS electron microscopy of N-PCR surface printed by 1.5 wt% and 2.5 wt% TiO_2_/PLA.

Figure 3 and Figure 4 show the SEM and EDS images of N-PCR fabricated by fused deposition modeling (FDM), respectively. It can be observed that FDM employs a layer-by-layer additive manufacturing approach, resulting in a relatively rough surface. The presence of TiO_2_ distributed within the TiO_2_/PLA-based PCR matrix is evident. As the proportion of TiO_2_ in the TiO_2_/PLA composite filament increases, the gaps in the N-PCR produced by FDM printing are significantly reduced, which may be attributed to the intermolecular interactions between TiO_2_ and PLA.

EDS analysis indicates that Ti elements are uniformly distributed in the N-PCR printed using 1.5 wt% and 2.5 wt% TiO_2_/PLA composite filaments. The homogeneous distribution is observed both between and within the layers of the PCRs. As the TiO_2_ content increases, the distribution of Ti elements becomes more concentrated.

### 2.2. Photocatalytic Performance of Different Reactors

The FRD-PCR was selected to investigate the effect of varying parameter C on the photocatalytic performance. As illustrated, the FRD-PCR with C = 0.3 exhibited photocatalytic degradation efficiencies of 76.4% (under rotational flow field) and 54.4% (under horizontal flow field) after 2.5 h, both of which were lower than those achieved by the FRD-PCR with C = 0.5, which reached 87.5% and 63.3% (Table 2), respectively. Furthermore, the C = 0.5 configuration demonstrated advantages in terms of material consumption, cost efficiency, and moderate operational stability. Therefore, for comparative analysis with other reactors, the FRD-PCR with a parameter C value of 0.5 was employed.

**Figure 5:**
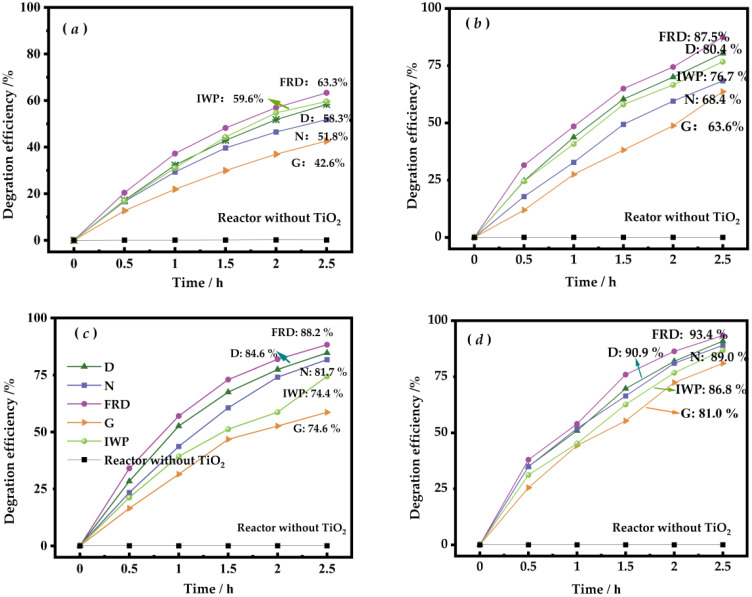
Degradation efficiency of TPMS PCRs printed by 1.5 wt% ((**a**): horizontal flow field; (**b**): rotational flow field), 2.5 wt% TiO_2_/PLA melt ((**c**): horizontal flow field; (**d**): rotational flow field) and reactor without TiO_2_.

Figure 5 demonstrates that the reactor without TiO_2_ exhibited no measurable photocatalytic activity under identical illumination conditions, confirming that the polymer substrate itself is photocatalytically inert. Figure 5a,b illustrate the photocatalytic degradation performance of FDM-printed 1.5 wt% TiO_2_/PLA-based TPMS-PCRs under horizontal and rotational flow fields, respectively. The photocatalytic degradation rate and overall efficiency were consistently lower under horizontal flow conditions compared to rotational flow conditions. Among the tested configurations, the G-PCR exhibited the lowest overall photocatalytic degradation rate (as indicated by its shallow slope) and the lowest degradation efficiencies (42.6% under horizontal flow and 63.6% under rotational flow). In contrast, the FRD-PCR demonstrated the highest degradation rate and efficiency, achieving 63.3% and 87.5% under horizontal and rotational flow fields, respectively. The D-PCR and IWP-PCR showed comparable photocatalytic degradation performances, with no significant difference between them.

Figure 5c,d depict the corresponding degradation results for structures printed with 2.5 wt% TiO_2_/PLA composite filaments. Increasing the TiO_2_ content led to improved degradation performance across all architectures. The FRD-PCR, which outperformed all other designs, reached a degradation efficiency of 93.4% in the rotational flow field. The N-PCR also exhibited a substantial enhancement in photocatalytic activity. Conversely, the G-PCR remained the least effective, with degradation efficiencies of 74.6% and 81.0% in the horizontal and rotational flow fields, respectively.

According to the literature [21], a reduction in the pore size of TPMS structures diminishes the optical penetration depth, whereas elevated porosity improves both light utilization efficiency and internal fluid flow dynamics. Consequently, the photocatalytic performance of TMPS-PCRs is co-determined by three key structural parameters: porosity, which governs light penetration and permeability; specific surface area, which dictates catalyst loading and active site availability; and average pore size, which influences mass transfer and light scattering behavior.

Table 3 summarizes the porosity, specific surface area, average pore size, and photocatalytic efficiency of TMPS-PCRs, using 2.5 wt% TiO_2_/PLA composites under rotational flow field conditions as an example, and provides an analysis of the corresponding structure–performance relationships. Meanwhile, in the design of TPMS structures, the parameter C serves as a fundamental mathematical variable controlling the geometric morphology by altering the isosurface threshold in the implicit function equation. It directly governs key structural properties including porosity, pore size distribution, and specific surface area. Increasing the value of C generally leads to reduced porosity and a more densely packed structure, whereas decreasing C promotes the formation of open porous networks with high permeability. This trend aligns closely with the photocatalytic performance observed in our study: the FRD, N and D structures with C = 0.5 showed higher apparent catalytic efficiency. These results demonstrate that tuning C enables synergistic optimization of light harvesting, mass transfer, and active site exposure, providing a critical theoretical basis for the design of high-efficiency catalytic substrates.

## 3. Materials and Methods

### 3.1. Synthesis of TiO_2_/PLA Composite Filament

The twin-screw 3D printing filament extruder was switched on and preheated for 30 min, with the temperature parameters for each processing zone set as detailed in Table 1. Once the actual temperatures of all temperature-controlled zones reached the preset values, the single-phase air-cooling system was activated. The extruder was initially purged with pure polylactic acid (PLA). The purging process was considered complete when the extrudate emerged as a milky-white, impurity-free semi-formed PLA filament.

**Figure 6:**
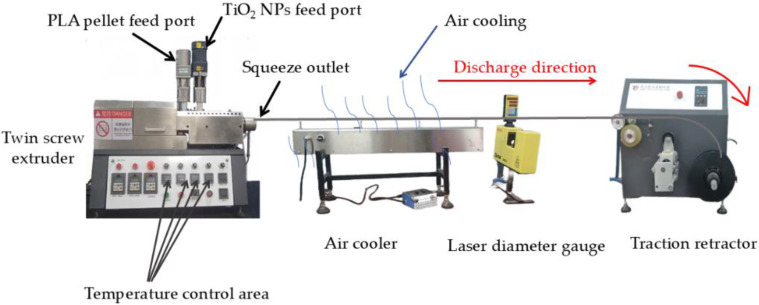
TiO_2_/PLA composite 3D printing wire production process.

Titanium dioxide nanoparticles (TiO_2_ NPs) and PLA raw pellets were pre-dried separately at 120 °C for 4 h. The TiO_2_ NPs were incorporated at mass fractions of 1.5% and 2.5%. After drying, the mixtures were gradually fed into the extruder through the feeding zone. The temperature profile of the twin-screw extruder was set as follows: Zone 1: 175 °C, Zone 2: 185 °C, Zone 3: 190 °C, Die Zone 1: 180 °C, and Die Zone 2: 175 °C. The composites were melt-blended at high temperatures and extruded through the discharge outlet. The extruded filament was cooled in an air-cooling chamber and then passed through a laser diameter gauge before being directed into the traction winder (Figure 6).

The winding speed was adjusted based on real-time diameter measurements from the laser gauge to synchronize with the extrusion rate, thereby ensuring the production of uniform cylindrical composite pellets. The filament was manufactured to meet the standard diameter of 1.75 mm for fused deposition modeling (FDM). The rotational speeds of the air-cooling chamber and the traction winder were maintained at 130 rad/min and 3.1 rad/min, respectively. Following the operational parameters of the twin-screw extruder outlined in Table 4, TiO_2_/PLA composite 3D printing filaments with varying ratios were successfully fabricated. Filament uniformity is critical to printing accuracy; therefore, the diameter deviation was controlled within ±0.05 mm.

### 3.2. Fabrication of Triply Periodic Minimal Surface (TPMS) Reactors via 3D Printing

The design of photocatalytic reactors must address several critical requirements: the catalyst support should possess a high specific surface area, achievable through hierarchical pore structures, nanocomposite materials, and customized 3D-printed carriers, to enhance active site density; light transmission and refractive index control must be optimized to minimize optical loss and improve photon utilization efficiency; the reactor should exhibit strong compatibility with light sources, ensuring that the emission spectrum aligns with the catalyst’s bandgap for improved energy efficiency and treatment performance; fluid channel configurations should enhance mass transfer to reduce diffusion resistance and avoid mass-transfer-limited reaction rates; lastly, sufficient loading stability and mechanical strength are essential to prevent frequent reactor failure.

Parameter C is a constant used to define the level set (isosurface) of the function F. Specifically, for a given value of C, the equation F(x, y, z) = C describes a surface in three-dimensional space. C is closely related to the volume fraction (or relative density) of the structure. TPMS structures allow precise control over the parameter C in their mathematical formulation, enabling high specific surface area, excellent light transmittance, high connectivity, structural stability, and light source adaptability—making them ideally suited for efficient photocatalytic reactor design. Taking the Gyroid surface as an example: when C = 0, the volume fraction is approximately 50%, meaning the solid and pore phases are equally proportioned; when C > 0, the volume fraction increases, resulting in a denser material; and when C < 0, the volume fraction decreases, yielding a more porous structure. Using a 3D printing plugin in Blender, TPMS models were designed and evaluated via geometric node analysis to compute key structural parameters. The main structural parameters corresponding to different values of C used in this study are summarized in Appendix A Table A1.

The parameter C strongly influences volume and porosity. It controls the amplitude of periodic variation in the TPMS: as C decreases, the structural “depressions” become shallower, the solid fraction increases, and the overall volume rises. Porosity is positively correlated with C: higher C values result in higher porosity, making them suitable for applications requiring high permeability or low density (e.g., filtration, lightweight materials).

The surface area of the FRD type decreases slightly with decreasing C, while other types (e.g., Gyroid, Diamond) exhibit an increase. Different TPMS mathematical expressions respond differently to changes in C: the N-type surface shows more pronounced fractal characteristics with increased wrinkling as C decreases. All types exhibit a decrease in specific surface area (S/V ratio) as C declines. Thus, high-C values are suitable for catalytic reactions (requiring high active site exposure), while low-C values are preferable for structural support (requiring high mechanical strength).

The G-type demonstrates balanced performance: at C = 1.2, the S/V ratio reaches 17.94 cm^2^/cm^3^ with a porosity of 89.56%, making it ideal for ultra-light porous supports or highly efficient adsorption applications. The N-type at C = 0.5 shows a porosity of 78.02%, higher than that of FRD (66.20%) and D-type (53.32%) under the same parameter, rendering it suitable for high-permeability fluid channel designs. The D-type exhibits low specific surface area characteristics: at C = 0.1, the S/V ratio is 8.79 cm^2^/cm^3^, representing a dense structure potentially applicable in load-bearing multifunctional components.

In summary, parameter C is a central variable for tuning TPMS structural properties. A decrease in C leads to increased volume, reduced porosity, and lower S/V ratio, though the sensitivity to C varies significantly among different structural types. Design should be tailored to specific applications (e.g., catalysis, filtration, lightweight structures) through optimization of C and selection of the appropriate TPMS type. The TPMS parameters and corresponding structural properties used in this study are listed in Table 5 below.

**Figure 7:**
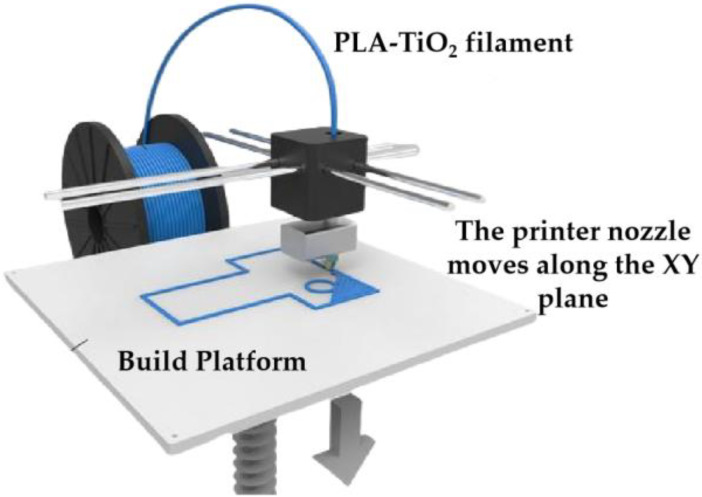
FDM 3D printing process.

**Figure 8:**
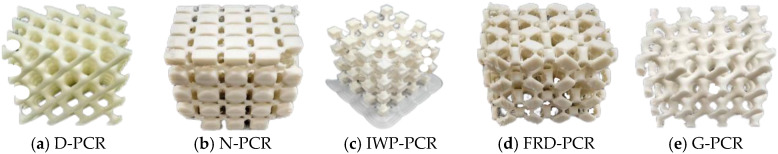
TPMS-PCR printing by TiO_2_/PLA melt deposition.

Using the FDM method (Figure 7), the models were printed with a layer height of 0.1 mm, 100% infill density, a nozzle temperature of 210 °C (slightly above the melting point of PLA), and a printing speed of 50 mm/s. Brim-type support was applied to ensure the stability of the bottom layers. As a result, triply periodic minimal surface structures with dimensions of 4 × 4 × 4 cm and containing 1.5 and 2.5 wt% TiO_2_ were successfully fabricated (Figure 8).

### 3.3. Photocatalytic Activity Test

**Figure 9:**
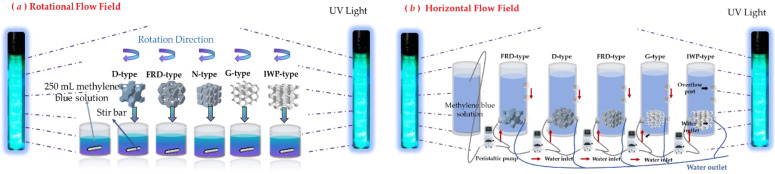
Two flow field performance tests ((**a**): rotational flow field; (**b**): horizontal flow field).

The photocatalytic activities of the five three-dimensional printed TPMS-PCRs (IWP, G, N, D, and FRD) were evaluated by monitoring the degradation of methylene blue (MB) under irradiation in two different flow field configurations (horizontal and rotational, as illustrated in Figure 9).

A 500 W mercury lamp (Shanghai Bilang Instrument Manufacturing Co., Ltd., Shanghai, China), providing a broad emission spectrum from 254 to 700 nm, served as the light source. The incident light power density on the reactor surface was measured to be 380 W/m^2^. The photocatalytic tests were conducted under the condition of a chilled water circulation system to maintain the internal temperature of the reactor at 20 °C. The target pollutant consisted of an aqueous methylene blue solution (MB, analytical grade, Sigma-Aldrich) at an initial concentration of 50 mg/L. Each experiment utilized a single monolithic TPMS-PCR structure as the catalyst.

Before each photocatalytic test, the MB solution was kept under continuous circulation or stirring in complete darkness for 5 h to achieve adsorption–desorption equilibrium between the dye and the catalyst surface, thereby ensuring that subsequent degradation resulted primarily from photocatalysis. After this dark adaptation period, the lamp was switched on to initiate the reaction. During irradiation, 3 mL aliquots were collected every 30 min over a total duration of 2.5 h.

For the horizontal flow field setup, a custom-made glass tube reactor (20 cm in height, 11 cm inner diameter) was used. The inlet and outlet ports (1 cm diameter each) were mounted horizontally 7 cm above the bottom and aligned with the center of the photocatalyst. A peristaltic pump (BW300+JZ15A, Baoding Dichuang Electronic Technology Co., Ltd., Baoding, China) circulated 500 mL of the MB solution through the reactor at a constant flow rate of 500 mL/min. In the rotational flow configuration, experiments were carried out in a standard glass vessel (10 cm height, 8 cm diameter) with the photocatalyst immobilized 3 cm above the bottom. A magnetic stirrer operating at 1200 rpm induced fluid motion with an average velocity of 0.5 m/s. The total solution volume in this configuration was 200 mL, and the vessel was positioned 2 cm from the light source.

All sampled aliquots were centrifuged at 10,000 rpm for 5 min to remove suspended particles prior to analysis. The absorbance of the clear supernatant was measured at 664 nm using an A360 UV-Vis spectrophotometer (Aoyi Instruments, Shanghai, China). The degradation efficiency (*η*, %) was calculated using Equation (1):

(1) $$\begin{array}{c} \eta =\frac{A_{t}-A_{0}}{A_{t}}\times 100\% \end{array}$$

where *A*_0_ is the absorbance after the dark adsorption period and before illumination, and *A_t_* is the absorbance at time *t*.

## 4. Conclusions and Outlook

In this study, TiO_2_/PLA as the supporting matrix to successfully fabricate composite filaments. Five types of TPMS-based photocatalytic reactors (TPMS-PCRs), namely N, D, FRD, G, and IWP structures, were manufactured via FDM 3D printing technology through modeling and slicing processes. This approach significantly improved the reusability of the photocatalytic reactors while reducing production costs.

With increasing TiO_2_ content, the reactors exhibited enhanced hydrophilicity, improving their wetting behavior and contact with the aqueous solution, thereby facilitating the catalytic degradation process. The triply periodic minimal surface (TPMS) porous architecture provided extended optical pathways and increased light reflection, greatly improving light utilization and activating more catalytic sites on the reactor surface.

The fabricated photocatalytic reactors demonstrated excellent degradation performance across different flow configurations. Among them, the FRD-type porous scaffold printed with 2.5 wt% TiO_2_/PLA composite filament exhibited the highest photocatalytic activity, achieving a methylene blue (MB) degradation rate of 93.4% within 2.5 h under rotational flow conditions, compared to 87.5% under horizontal flow conditions.

Furthermore, the factors influencing photocatalytic efficiency were systematically discussed, leading to the conclusion that the photocatalytic performance of the TMPS-PCRs is co-determined by three key structural parameters: porosity, specific surface area, and average pore size.

Although this study has successfully demonstrated the fabrication and enhanced performance of TiO_2_/PLA-based TPMS photocatalytic reactors (TPMS-PCRs) produced via FDM, the role of nanoscale properties in influencing photocatalytic behavior remains incompletely understood. The high-temperature shear conditions during printing, as well as the interfacial characteristics between PLA and TiO_2_, may significantly impact nanoparticle dispersion, surface chemistry, and topological features.

To advance both the fundamental knowledge and practical deployment of these structured photocatalysts, subsequent research will focus on utilizing advanced characterization techniques, such as XPS and TEM, to quantitatively evaluate how the printing process affects the surface properties of the nanocatalysts and elucidating the mechanisms through which PLA-TiO_2_ interfacial interactions modulate the electronic structure and charge separation efficiency of the composite catalytic system.

## Figures and Tables

**Table 1:** Contact angle test data.

Sample	Left contact Angle/°	Right Contact Angle/°
Pure PLA	80.771	80.771
1.5 wt% TiO_2_/PLA	62.587	62.587
2.5 wt% TiO_2_/PLA	62.301	62.301

**Table 2:** Effect of parameter C on the photocatalytic degradation efficiency of FRD-PCRs.

Parameter C	Photocatalytic Degradation Efficiency After 2.5 h	
	(1.5 wt% TiO_2_/PLA, Rotational Flow Field)	(1.5 wt% TiO_2_/PLA, Horizontal Flow Field)
0.5	87.5%	63.3%
0.3	76.4%	54.4%

**Table 3:** The relationship of porosity, specific surface area, average pore size, and photocatalytic efficiency.

TPMS Type	Parameter C	Porosity (%)	Specific Surface Area (cm^2^/cm^3^)	Average Pore Size (mm)	Photocatalytic Efficiency	Interpretation
FRD	0.5	66.20%	12.47	8.41	93.4%	The largest pore size provides excellent mass transfer, enabling rapid diffusion of reactants/products. Although porosity and specific surface area are moderate, its performance is superior under rotational flow conditions due to enhanced convective transport.
N	0.5	78.02%	14.17	6.74	89.0%	A balanced structure with relatively high porosity and specific surface area, combined with a moderate pore size, achieves an optimal trade-off among light harvesting, mass transfer, and active site availability
D	0.5	53.32%	9.86	5.1	90.9%	Possesses the lowest porosity, specific surface area, and smallest pore size. Although typically associated with inferior performance, its high efficiency here may be attributed to unique pore connectivity and optimized light distribution under specific flow conditions (e.g., low velocity).
G	1.2	89.56%	17.94	7.89	81.0%	Exhibits the highest porosity and specific surface area, which facilitate light transmission and catalyst loading. However, the relatively large pore size may lead to insufficient light utilization (e.g., partial light penetration without reaction) or non-uniform flow distribution, resulting in sub-optimal efficiency.
IWP	2.0	75.12%	11.57	7.83	86.8%	Displays well-balanced properties. The large pore size enhances mass transfer, but the relatively low specific surface area limits the number of reactive sites.

**Table 4:** Main parameters of twin-screw extruder.

Feed Port Speed (rad/min)		Heating Zone Temperature Control (°C)			Discharge Port Temperature Control (°C)	
(Pellet) Feed Port Speed	(Powder) Feed Port Speed	Zone 1	Zone 2	Zone 3	Discharge Zone 1	Discharge Zone 2
10	8	175	185	190	180	175

**Table 5:** Selected TPMS parameters and corresponding main structural parameters (4 cm × 4 cm × 4 cm).

TPMS	Parameter C	Volume (cm^3^)	Surface Area (cm^2^)	Porosity (%)	Specific Surface Area (cm^2^/cm^3^)
FRD	0.5	20.2806	252.8271	66.20%	12.47
	0.3	28.1025	262.088	53.16%	9.33
N	0.5	28.0057	276.1458	78.02%	14.17
D	0.5	13.1894	186.8994	53.32%	9.86
G	1.2	6.265	112.3928	89.56%	17.94
IWP	2.0	14.9278	172.6605	75.12%	11.57

## Data Availability

The original contributions presented in this study are included in the article. Further inquiries can be directed to the corresponding author.

## Abbreviations

The following abbreviations are used in this manuscript:

TPMS	Triply Periodic Minimal Surface
PCR	Photocatalytic Reactors
FRD	Fischer-Radin-Dunn
N	Neovius
D	Diamond
IWP	I-graph Wrapped Package
G	Gyroid

## Appendix A

Table A1 summarizes the principal structural parameters of the 4 cm × 4 cm × 4 cm TPMS-based photocatalytic reactors corresponding to varying design parameter C values.

**Table A1:** Main structural parameters corresponding to different design parameter C values (4 cm × 4 cm × 4 cm).

TPMS	Parameter C	Volume (cm^3^)	Surface Area (cm^2^)	Poosity (%)	Specific Surface Area (cm^2^/cm^3^)
FRD	0.5	20.2806	252.8271	66.20%	12.47
	0.4	24.3341	263.4307	59.44%	10.83
	0.3	28.1025	262.088	53.16%	9.33
	0.2	31.5176	260.35	47.47%	8.26
	0.1	34.7453	254.8902	42.09%	7.34
N	0.5	28.0057	276.1458	78.02%	14.17
	0.4	29.399	283.1603	71.65%	12.12
	0.3	30.7664	288.2382	65.40%	10.62
	0.2	32.1576	292.0641	59.25%	9.46
	0.1	33.5395	294.8173	53.14%	8.51
D	0.5	13.1894	186.8994	53.32%	9.86
	0.4	17.0088	206.1038	51.00%	9.63
	0.3	20.758	220.4739	48.72%	9.37
	0.2	24.453	231.291	46.40%	9.08
	0.1	28.114	239.2879	44.10%	8.79
G	1.2	6.265	112.3928	89.56%	7.94
	1	10.8215	141.5935	81.96%	13.08
	0.8	15.175	161.8086	74.71%	10.66
	0.6	19.4092	177.3254	67.65%	9.14
IWP	2.0	14.9278	172.6605	75.12%	1.57
	1.8	16.9145	181.4704	71.81%	10.73
	1.6	18.8147	188.9283	68.64%	10.04

Table A2 shows relative crystallinity of the reactor printed using 2.5 wt% and 1.5 wt% TiO_2_/PLA.

**Table A2:** XRD phases and relative crystallinity of the reactor printed using 2.5 wt% and 1.5 wt% TiO_2_/PLA.

Samples	Phases	Relative Crystallinity (%)
The reactor printed using 2.5 wt% TiO_2_/PLA	TiO_2_	2.3
	PLA	97.7
The reactor printed using 1.5 wt% TiO_2_/PLA	TiO_2_	1.4
	PLA	98.6
